# A Comparative Analysis of Deaths Due to Drug‐Related Causes Between Rural and Urban Areas in the United Kingdom, 1997–2026

**DOI:** 10.1111/dar.70285

**Published:** 2026-10-28

**Authors:** Emmert Roberts, Jürgen Rehm, Caroline Copeland

**Affiliations:** ^1^ National Addiction Centre King's College London London UK; ^2^ South London and the Maudsley NHS Foundation Trust London UK; ^3^ Institute for Mental Health Policy Research Centre for Addiction and Mental Health Toronto Canada; ^4^ Campbell Family Mental Health Research Institute Centre for Addiction and Mental Health Toronto Canada; ^5^ Dalla Lana School of Public Health University of Toronto Toronto Canada; ^6^ Department of Psychiatry University of Toronto Toronto Canada; ^7^ Faculty of Medicine, Institute of Medical Science University of Toronto Toronto Canada; ^8^ Centre for Interdisciplinary Addiction Research, Department of Psychiatry and Psychotherapy University Medical Centre Hamburg‐Eppendorf Hamburg Germany; ^9^ Program on Substance Abuse and World Health Organization Collaborating Centre Public Health Agency of Catalonia Barcelona Spain; ^10^ Faculty of Life Sciences & Medicine King's College London London UK

**Keywords:** demography, drug‐related deaths, epidemiology, opioids, rural, urban

## Abstract

**Introduction:**

Multiple jurisdictions report disproportionately elevated rates of drug‐related harm in urban settings, alongside unique treatment needs in rural populations. We examined differences in case characteristics, circumstances of death and implicated drugs among individuals dying from drug‐related causes, stratified by rural or urban setting, and determined changes in the proportion of deaths between settings over time.

**Methods:**

Retrospective registry‐based analysis of coronial records of individuals who died from drug‐related causes, submitted to the National Programme on Substance Use Mortality in the UK, 1997–2026. UK Government Rural–Urban Classification methodology dichotomised cases using postcode at date of death.

**Results:**

Overall, 39,975 decedents died, 36,350 (90.9%) in urban settings. The urban percentage decreased significantly over the study period from 96.6% in 1999 to 92.3% in 2025 (AAPC −0.34%, 95% CI −0.47% to −0.15%, *p* = 0.002). Accidental poisoning was the most common direct cause of death in both rural (45.5%) and urban (54.8%) settings, while intentional self‐poisoning was more common rurally (17.3% vs. 9.5%). Opioids were the most frequently implicated drug group in both rural (61.1%) and urban (65.4%) settings. The median number of drugs implicated did not differ significantly between settings (both 2, IQR 1–3), but rural decedents had a significantly higher median number of prescribed implicated drugs (1, IQR 0–2 vs. 0, IQR 0–2).

**Discussion and Conclusions:**

Over three decades, approximately one in 11 UK drug‐related deaths occurred rurally. Intentional self‐poisoning was substantially more common in rural fatalities, and opioids were the leading implicated drug group across both settings.

## 1 Introduction

In the last decade, many countries have reported their highest number of drug‐attributable deaths since records began [1]. Multiple studies have historically reported disproportionately elevated rates of some drug‐related harms in urban areas while simultaneously identifying unique drug use profiles and treatment needs in rural populations [2, 3]. Despite this, few national or regional jurisdictions routinely report individual drug‐related harms by granular area level metrics or provide data enabling comparison between rural and urban areas [3]. This can lead to challenges in understanding specific patterns of harm, assessment of changing trends over time, and any appropriate tailoring of preventative interventions [3].

The United Kingdom Government Office for National Statistics (ONS) publishes annual data on deaths due to drug poisoning, with breakdowns across 11 regions [4]. However, unlike other ONS health outputs (e.g., Characteristics of women with an endometriosis diagnosis in England) [5], these publications do not typically report data enabling the examination of rural/urban differences, nor are they able to provide granular detail relating to the circumstances of death or individual implicated drugs [6]. While previous epidemiological studies have examined inequities in UK drug‐related harms by local area indices of multiple deprivation [7], to our knowledge, none have explicitly examined differences in rural and urban drug‐related deaths. Several international jurisdictions have recognised the need to adapt both rural and urban drug treatment service delivery in response to observed differential profiles of drug‐related harms [3]. Understanding the specific case characteristics and/or particular drug use profiles associated with fatalities in rural/urban areas, and if this has changed over time, could therefore aid in identifying structural factors driving any observed differences and lead to the development of more targeted treatment models or specific harm reduction advice.

To address these gaps, the study aimed to determine: (i) the case characteristics and sociodemographics; (ii) the circumstances of death; and (iii) the number and type of drugs implicated in deaths among people dying due to drug‐related causes stratified by if death occurred in a rural or urban area in the UK. The study additionally aimed to determine any changes in the proportion of deaths occurring in rural or urban areas over time.

## 2 Methods

This study is reported according to the Strengthening the Reporting of Observational Studies in Epidemiology (STROBE) statement [8], the completed checklist is available as Table S1.

### 2.1 The National Programme on Substance Use Mortality

We conducted a retrospective registry‐based analysis using records drawn from the National Programme on Substance Use Mortality (NPSUM). This paper contains recycled methods text material from previous NPSUM publications and is reported in accordance with guidance from the text recycling research project (https://textrecycling.org) [9]. The NPSUM database contains information on an observational cohort that collects coronial data on deaths at any age related to any psychoactive drug, other than caffeine or where the sole substance implicated was alcohol or nicotine [9]. In total, NPSUM holds records on more than 58,000 deaths with reports received from England and Wales, the Channel Islands and the Isle of Man since 1997. Additional reports were received from the Scottish Crime and Drug Enforcement Agency between 2004 and 2011 and from the General Register Office for Northern Ireland since 2004, with these deaths included within the full NPSUM cohort [10]. Coronial files typically include the coroner's decision as to cause of death, statements from witnesses, family and friends, first responders, general practitioner and hospital records which may include documentation of formal diagnoses and drug use history. Toxicology is discretionally requested by individual coroners, and coronial jurisdictions choose to voluntarily report all deaths within their area to NPSUM if any of the following criteria are met: (i) psychoactive substance(s) are directly implicated in death; (ii) an individual has a history of dependence or problematic use of drugs; or (iii) if controlled drugs are identified at post‐mortem.

The King's College London Biomedical and Health Sciences, Dentistry, Medicine and Natural and Mathematical Sciences Research Ethics Subcommittee re‐confirmed in August 2025 that NPSUM does not require Research Ethics Committee review as all individuals are deceased.

### 2.2 Case Categorisation

The UK Government ONS reports an official Rural Urban Classification (RUC) which provides a consistent and standardised statistical method for the binary categorisation of postcode areas as either rural or urban (based on address density, physical settlement form, population size and relative access to major towns and cities) [11, 12]. This categorisation methodology applies only to UK mainland postcodes (i.e., those in England, Scotland or Wales) with the 2021 census demonstrating 17.1% of this population resided in rural areas, this having marginally reduced over the previous two decades, estimated at 17.6% in 2011 and 19.4% in 2001 [12]. NPSUM contains a field which reports the postcode at the decedent's place of death. All cases with an available full or partial UK mainland postcode reported to NPSUM between 1997 and 1 January 2026 (*n* = 40,399) were eligible for inclusion. NPSUM postcodes were categorised as rural or urban via linkage to the latest version (February 2026) of the ONS Postcode Directory, using the 2021 census taxonomy to distinguish urban (RUC codes UN1, UF1) from rural areas (RSN1, RSF1, RLN1, RLF1) [12]. Where 2021 codes were unavailable, due to some older postcodes having been retired, numeric 2011 RUC census codes (1–2 urban; 3–6 rural) were applied.

Full postcodes were matched in the first instance (Number of records successfully matched = 39,127/40,399 (96.8%)). Unmatched postcodes were subsequently subject to a sequential linkage procedure: (i) standardisation of formatting by removing any extraneous whitespace and reinserting spaces in standard postcode positions before re‐linking (*n* = 413/40,399 (1.0%)); (ii) where records reported only a partial postcode linkage was facilitated against a district‐level RUC lookup derived from the ONS Postcode Directory (*n* = 421/40,399 (1.0%)); (iii) manual review and correction of any remaining identifiable transcription errors (*n* = 14/40,399 (0.03%)). A total of 424 records (1.1%) remained unmatched after all stages.

### 2.3 Measures

Data on decedent sociodemographics (including age, sex, ethnicity, employment status etc.), mental health history (i.e., a documented history of individual mental health conditions), substance use history (i.e., documented history of substance dependence and injecting status), toxicology and which individual substances were ultimately deemed implicated in death were extracted from coronial files. All cases additionally recorded findings concerning the single disease or condition that led directly to death, any antecedent cause(s) and other significant conditions contributory to death.

### 2.4 Statistical Analysis

We generated descriptive statistics for all decedents. Means, standard deviations (SD) and ranges were presented for continuous variables, as well as median and interquartile range (IQR) in skewed distributions. Overall percentages were reported for all binary and categorical variables and the valid percentage (i.e., the percentage of cases where history was available) was reported for mental health and substance use variables. Continuous variables were compared using Welch's unpaired *t*‐test, ordinal and count variables using the Wilcoxon rank sum test and categorical variables using a chi‐squared test [13]. Analyses were conducted in STATA IC version 16 with the significance level set at 0.05. We used the Joinpoint Regression Program, version 5.4.1 (US Statistical Research and Applications Branch, National Cancer Institute), to estimate the average annual percent change in the proportion of deaths that occurred in an urban area across the studied time period. A log linear model used weighted Bayesian information criteria estimates to determine best model fit. Autocorrelation parameters were assessed with errors found to be uncorrelated. The analysis plan was not pre‐registered and the results should be considered exploratory.

## 3 Results

A total of 39,975 decedents in the NPSUM database had a valid matched postcode, in England, Scotland or Wales, recorded at their place of death between 1997 and 2026. In 3625 (9.1%) cases death occurred in a rural postcode area compared to 36,350 (90.9%) in an urban postcode area. A two‐segment Joinpoint model best fit the data with a statistically significant reduction in the percentage of deaths due to drug‐related causes occurring in an urban area across the studied time‐period (average annual percent change −0.34% 95% confidence interval −0.47% to −0.15%, *p* = 0.002). This was driven by a reduction from 96.6% in 1999 to 91.5% in 2006 (annual percentage change −1.17%, −1.83% to −0.50%, *p* = 0.001), with no change observed between 2006 (91.5%) and 2025 (92.3%) (annual percentage change −0.04%, −0.13% to 0.06%, *p* = 0.41). The overall trend and full two‐segment Joinpoint regression model is available as Figure S1.

### 3.1 Sociodemographics and Case Characteristics

Sociodemographic and case characteristics of all decedents stratified by if death occurred in a rural or urban area can be found in Table 1. Overall, decedents were predominantly male (*n* = 28,998, 72.5%), of White ethnicity (*n* = 23,477, 58.7%) and had a mean age of 42.5 years (SD 13.2; range 1–105). When compared to rural areas, there was a higher percentage of fatalities in urban areas in which decedents were male (72.9% vs. 69.4%; *χ* ^2^ 19.6, *p* < 0.001). There was an overall difference in decedent ethnicity (*χ* ^2^ 34.2, *p* < 0.001), driven largely by a lower percentage of fatalities among White decedents in urban areas (58.5% vs. 61.2%). There was an overall difference in decedent employment status (*χ* ^2^ 181.3, *p <* 0.001), driven largely by a higher percentage of fatalities in urban areas where decedents were either unemployed (42.4% vs. 36.3%) or employed in non‐manual professions (16.9% vs. 8.3%).

**TABLE 1:** Sociodemographic, case characteristics, circumstances of death and mental health and substance use history of people dying due to drug‐related causes stratified by if death occurred in a rural or urban area in the United Kingdom, 1997–2026.

		All (*n* = 39,975) *n* (%)	Rural (*n* = 3625) *n* (%)	Urban (*n* = 36,350) *n* (%)	*p*			All (*n* = 39,975) *n* (%)	Rural (*n* = 3625) *n* (%)	Urban (*n* = 36,350) *n* (%)	*p*
Age	Mean, years	42.5 (SD 13.2; Range 1–105)	43.8 (SD 14.6; Range 1–98)	42.3 (SD 13.1; Range 1–105)	< 0.001	Direct cause of death (ICD‐10 code)	Poisoning, accidental (X40‐X45)	21,554 (53.9)	1648 (45.5)	19,906 (54.8)	< 0.001
Sex	Female	10,977 (27.5)	1109 (30.6)	9868 (27.1)	< 0.001		Poisoning, intentional (X60‐X84)	4078 (10.2)	628 (17.3)	3450 (9.5)	
	Male	28,998 (72.5)	2516 (69.4)	26,482 (72.9)			Poisoning, undetermined intent (T40.5, Y10‐Y14)	3354 (8.4)	314 (8.6)	3040 (8.4)	
Ethnicity	White	23,477 (58.7)	2220 (61.2)	21,257 (58.5)	< 0.001		Cardiovascular pathology (e.g., I21, I49 etc.)	1216 (3.0)	157 (4.3)	1059 (2.9)	
	Black	335 (0.9)	13 (0.4)	322 (0.9)			Respiratory pathology (e.g., J18, J96 etc.)	4118 (10.3)	419 (11.5)	3699 (10.2)	
	Asian	266 (0.7)	5 (0.1)	261 (0.7)			Other (e.g., T17, K72 etc.)	5655 (14.1)	459 (12.6)	5196 (14.3)	
	Unknown/not recorded	15,897 (39.8)	1387 (38.3)	14,510 (39.9)		Mental health^c^	People with a history of any mental health disorder	9778/10,181 (96.0)	970/1006 (96.4)	8808/9175 (96.0)	0.52
Employment status^a^	Employed (manual)	6492 (16.2)	663 (18.3)	5829 (16.0)	< 0.001		People with a history of a depressive disorder	5896/10,181 (57.9)	623/1006 (f61.9)	5273/9175 (57.5)	0.01
	Employed (Non‐manual)	2819 (7.1)	299 (8.3)	2520 (16.9)			People with a history of schizophrenia	1003/10,181 (9.8)	56/1006 (5.5)	947/9175 (10.3)	< 0.001
	Unemployed	16,721 (41.8)	1317 (36.3)	15,404 (42.4)			People with a history of bipolar affective disorder	533/10,181 (5.2)	55/1006 (5.5)	478/9175 (5.2)	0.73
	Self‐employed	618 (1.6)	99 (2.7)	519 (1.4)			People with a history of an anxiety disorder	2551/10,181 (25.1)	279/1006 (27.7)	2272/9175 (24.8)	0.04
	Retired/invalid/sickness	5215 (13.1)	647 (17.8)	4568 (12.5)		Addiction^c^, ^d^	People with a history of substance dependence	21,642/29,165 (74.2)	1617/2661 (60.8)	20,025/26,504 (75.6)	< 0.001
	Unknown	8110 (20.3)	600 (16.6)	7510 (20.7)			People with an history of injecting behaviour	4713/8947 (52.7)	301/915 (32.9)	4412/8032 (54.9)	< 0.001
Year of death^b^	1997–2000	242 (0.6)	6 (0.2)	236 (0.7)	< 0.001						
	2001–2005	3623 (9.1)	261 (7.2)	2362 (9.3)							
	2005–2010	8510 (21.3)	747 (20.6)	7763 (21.4)							
	2011–2015	6793 (17.0)	626 (17.3)	6167 (17.0)							
	2016–2020	8641 (21.6)	856 (23.6)	7785 (21.4)							
	2021–2026	12,163 (30.4)	1129 (31.1)	11,034 (30.4)							

Abbreviation: SD, standard deviation.

^a^ Definitions based on those used by the United Kingdom Office of National Statistics [14].

^b^ To January 2026.

^c^ Valid percentage reported (i.e., the percentage of cases where history was available).

^d^ Addiction here refers to the number of people with a history of substance dependence and the number of people with an history of injecting behaviour.

### 3.2 Circumstances of Death and Mental Health and Substance Use History

Circumstances of death and mental health and substance use histories can be found in Table 1. Poisoning was the predominant disease or condition that was certified as the direct cause of death with 45.5% (*n* = 1648) of deaths in rural areas directly due to accidental poisoning (ICD‐10 codes, X40—X45) and 54.8% (*n* = 19,906) of deaths in urban areas. Comparatively, 17.3% (*n* = 628) of deaths in rural areas were directly due to intentional self‐poisoning (ICD‐10, X60‐X84) relative to 9.5% (*n* = 3450) in urban areas. In cases where medical history was available, there was a higher percentage of fatalities in urban areas compared to rural areas in which decedents had a documented history of schizophrenia (10.3% vs. 5.5%; *χ* ^2^ 23.1, *p* < 0.001), substance dependence (75.6% vs. 60.8%, *χ* ^2^ 276.3, *p* < 0.001) or injecting behaviour (54.9% vs. 32.9%, χ^2^ 160.0, *p* < 0.001) and a lower percentage with a documented history of an anxiety disorder (24.8% vs. 27.7%, *χ* ^2^ 4.3, *p* = 0.039).

### 3.3 Number and Type of Drugs Implicated in Death

The number and type of drugs implicated in death can be found in Table 2. There was no statistically significant difference between rural and urban areas in the median overall number of drugs implicated in death (2, IQR 1, 3 vs. 2, IQR 1, 3, *p* = 0.3), however there was a higher median number of drugs for which the decedent had a valid prescription implicated in death in rural areas (1, IQR 0, 2 vs. 0, IQR 0, 2, *p* < 0.001). When compared to rural areas, there was a higher percentage of fatalities in urban areas in which multiple (i.e., ≥ 2) substances were implicated in death (57.0% vs. 54.5%, *χ* ^2^ 8.5, *p* = 0.004).

**TABLE 2:** The number and type of drugs implicated in death as deemed by the coroner among people dying due to drug‐related causes stratified by if death occurred in a rural or urban area in the United Kingdom, 1997–2026.

Drug implicated in death		All (*n* = 39,975) *n* (%)	Rural (*n* = 3625) *n* (%)	Urban (*n* = 36,350) *n* (%)	*p*
All	Median number of drugs implicated	2 (IQR 1, 3; range 1–13)	2 (IQR 1, 3; range 1–13)	2 (IQR 1, 3; range 1–13)	0.3
	Only single substance implicated	17,283 (43.2)	1650 (45.5)	15,633 (43.0)	0.004
	Multiple substances implicated	22,692 (56.8)	1975 (54.5)	20,717 (57.0)	
Prescribed	Median number of prescribed drugs implicated	0 (IQR 0, 2; range 1–10)	1 (IQR 0, 2; range 1–10)	0 (IQR 0, 2; range 1–10)	< 0.001
	Any prescribed drug implicated	19,727 (49.3)	1894 (52.2)	17,833 (49.1)	< 0.001
Opioids	Any opioid	26,001 (65.0)	2213 (61.1)	23,788 (65.4)	< 0.001
	Heroin	16,098 (40.3)	1197 (33.0)	14,901 (41.0)	< 0.001
	Methadone	7956 (19.9)	559 (15.4)	7397 (20.4)	< 0.001
	Codeine	3000 (7.5)	326 (8.9)	2674 (7.4)	< 0.001
	Buprenorphine	465 (1.7)	38 (1.1)	427 (1.2)	0.50
Gabapentinoids	Any gabapentinoid	4388 (11.0)	400 (11.0)	3988 (11.0)	0.91
Benzodiazepines	Any benzodiazepine	8457 (21.2)	850 (23.5)	7607 (20.9)	< 0.001
	Diazepam	5009 (12.5)	439 (12.1)	4570 (12.6)	0.42
Antidepressants	Any antidepressant	6777 (17.0)	738 (20.4)	6039 (16.6)	< 0.001
Cannabis	Cannabis	544 (1.4)	46 (1.3)	498 (1.4)	0.62
Synthetic cannabinoid receptor agonists	Any SCRA	343 (0.9)	16 (0.4)	327 (0.9)	0.004
Cocaine	Cocaine	7763 (19.4)	539 (14.7)	7224 (19.9)	< 0.001
Alcohol^a^	Alcohol	8683 (21.7)	710 (19.6)	7973 (21.9)	0.001
MDMA^b^	MDMA	774 (1.9)	73 (2.0)	701 (1.9)	0.72
Ketamine	Ketamine	436 (1.1)	61 (1.7)	375 (1.0)	< 0.001

Abbreviations: IQR, interquartile range; SCRA, synthetic cannabinoid receptor agonist; SD, standard deviation.

^a^ Within NPSUM deaths in which alcohol is the only substance detected at post‐mortem are not recorded. As such, all drug‐related deaths in which alcohol is implicated in NPSUM have, by definition, multiple substances implicated.

^b^ 3,4‐methylenedioxymethamphetamine (ecstasy).

Opioids were the most commonly implicated drug group in both rural (*n* = 2213, 61.1%) and urban (*n* = 23,788, 65.4%) areas. When compared to rural areas, there was a higher percentage of fatalities in urban areas which implicated any opioid (65.4% vs. 61.1%, *χ* ^2^ 28.0, *p* < 0.001), heroin (41.0% vs. 33.0%, *χ* ^2^ 87.1, *p* < 0.001), methadone (20.4% vs. 15.4%, *χ* ^2^ 50.2, *p* < 0.001), cocaine (19.9% vs. 14.7%, *χ* ^2^ 52.8, *p* < 0.001), alcohol (21.9% vs. 19.6%, *χ* ^2^ 10.7, *p* = 0.001) and any synthetic cannabinoid receptor agonist (0.9% vs. 0.4%, *χ* ^2^ 8.1, *p* = 0.004). Conversely, there was a lower percentage of fatalities which implicated codeine (7.4% vs. 8.9%, *χ* ^2^ 12.7, *p* < 0.001), any benzodiazepine (20.9% vs. 23.5%, *χ* ^2^ 12.6, *p* < 0.001), any antidepressant (16.6% vs. 20.4%, *χ* ^2^ 32.8, *p* < 0.001) and ketamine (1.0% vs. 1.7%, *χ* ^2^ 13.0, *p* < 0.001).

## 4 Discussion

Over the last three decades within the NPSUM cohort approximately one in every 11 deaths due to a drug‐related cause occurred in a rural area, with evidence demonstrating an increase in the proportion of deaths occurring in rural areas over time. There were lower percentages of male, ethnically White and unemployed decedents in rural areas, alongside fewer individuals with documented diagnoses of schizophrenia, substance dependence and injecting behaviour.

Consistent with previous studies, accidental poisoning was the most common direct cause of death, with over two fifths of fatalities in both areas attributable to this preventable cause [3, 15]. However, a substantially higher percentage of fatalities in rural areas were directly due to intentional self‐poisoning. Previous literature highlights elevated rates of suicide within rural communities, including in the UK [16], typically describing increased access to high lethality means such as firearms or poisonous chemicals as associated factors [17], but the lower density of healthcare may also play a role [18]. This study provides evidence that intentional self‐poisoning also represents a significant burden, within deaths due to drug use, in rural populations and provides advocacy to ensure the adequate exploration of mental illness and suicidality during rural drug service and wider healthcare assessments [19].

Notably, in rural areas there was a significant increase in the median number of prescribed medications implicated in death with a significantly higher number of fatalities in which benzodiazepines, antidepressants, and codeine were implicated. Prescribing practice, dispensing and the formal supervision of prescribed medication consumption may differ within rural settings, and, within the UK, it is common for rural primary care practices to concurrently dispense medications and provide pharmacy services on site, often without independent pharmacist oversight [20]. Rural prescribers need to be aware of the overrepresentation of these substances in deaths following drug use and ensure that adequate consideration is given to dose, length of prescription and the potential need for supervised consumption.

The majority of deaths in both areas implicated multiple substances, suggesting that the nationally observed trend of increasing prevalence of polysubstance use is not isolated to urban communities [4]. Additionally, opioids remained the drug group implicated in the majority of fatalities in both areas, serving to reinforce the need to ensure the delivery of high‐quality interventions for the management of opioid use disorder, including the provision of appropriately dosed opioid agonist therapy and the provision of take‐home naloxone. Both of these interventions may face unique logistical challenges in rural settings, but they remain the interventions most able to reduce opioid‐related mortality [3].

The study has several strengths including the granular examination of substances implicated in death and the large national time frame. There are, however, several important limitations of NPSUM, particularly that variables of potential interest may either not be available or may be incomplete (e.g., over a third of cases contain no reported ethnicity) [9]. A substantial number of cases did not report a valid UK mainland full or partial postcode at place of death (*n* = 13,212) and were therefore unable to be matched and classified as deaths in rural or urban areas. However, reassuringly post hoc analyses revealed no statistically significant differences in studied variables, other than decedent employment status, between this population and the population with a valid full or partial postcode, available as Table S2. Due to the voluntary submission nature, NPSUM has incomplete coverage of the UK coronial system. Although over 90% of coroners report, non‐reporting jurisdictions contain heavily populated areas with previous triangulation work estimating that NPSUM receives between 65%–70% of all drug poisoning deaths officially reported by the ONS [21]. There is also variability of toxicological examination across cases, thus some deaths will likely have been missed and some implicated substances not identified. While the overall numbers of deaths reported should therefore be taken as conservative estimates, NPSUM remains the only data source currently able to granularly identify individual substances implicated in death at the individual postcode level across some regions of the UK. Use of district‐level postcode matching additionally has the potential to introduce rural/urban misclassification; however, the potential impact is minimal given only 1% of records were matched via district‐level look up.

In conclusion, a substantial proportion of deaths due to drug‐related causes occur in rural areas throughout the UK. Opioids were the drug group implicated in the highest proportion of deaths in both rural and urban areas and ensuring access to timely, evidence‐based interventions for the diagnosis and management of opioid use disorder should be a priority. Professionals and people who use drugs in rural areas should be particularly aware of the high proportion of deaths due to intentional self‐poisoning and the overrepresentation of prescribed medications, benzodiazepines, antidepressants and codeine.

## Author Contributions

Each author certifies that their contribution to this work meets the standards of the International Committee of Medical Journal Editors. **Emmert Roberts:** conceptualisation (equal); formal analysis (lead); funding acquisition (lead); investigation (lead); methodology (lead); project administration (lead); resources (equal); software (equal); writing – original draft (lead). **Jürgen Rehm:** investigation (equal); supervision (equal); validation (equal); writing – review and editing (equal). **Caroline Copeland:** conceptualisation (equal); data curation (lead); investigation (equal); methodology (equal); project administration (equal); resources (equal); supervision (equal); validation (equal); writing – review and editing (equal).

## Funding

This study was supported by the corresponding author's National Institute of Health and Care Research Advanced Fellowship (NIHR302215) and a Royal Society of Medicine Ellison‐Cliffe Travelling Fellowship. JR was supported by the Canadian Institutes of Health Research (FRN‐181677). The funders had no contribution to the study design; the collection, analysis and interpretation of data; the writing of the report; and the decision to submit the article for publication.

## Conflicts of Interest

All authors have completed the ICJME Unified conflicts of interest form (available on request from the corresponding author) and declare: no support from any organisation for the submitted work; no financial relationships with organisations that might have an interest in the submitted work in the previous 3 years, and no other relationships or activities that could appear to have influenced the submitted work.

## Supporting information


**Figure S1:** The proportion of deaths per year due to drug‐related causes that occurred in an urban setting in the United Kingdom, 1997–2026.
**Table S1:** Strengthening the Reporting of Observational Studies in Epidemiology (STROBE) statement checklist.
**Table S2:** Sociodemographic, case characteristics, circumstances of death and mental health and substance use history of people dying due to drug‐related causes stratified by if cases reported a valid postcode at the decedent's place of death in the United Kingdom, 1997–2026.

## Data Availability

The data that support the findings of this study are available on request from the corresponding author. The data are not publicly available due to privacy or ethical restrictions.

## References

[1] L. Degenhardt , F. Charlson , A. Ferrari , et al., “The Global Burden of Disease Attributable to Alcohol and Drug Use in 195 Countries and Territories, 1990–2016: A Systematic Analysis for the Global Burden of Disease Study 2016,” Lancet Psychiatry 5, no. 12 (2018): 987–1012. 10.1016/S2215-0366(18)30337-7 PMC6251968 30392731

[2] C. B. Oser , C. G. Leukefeld , M. Staton Tindall , et al., “Rural Drug Users: Factors Associated With Substance Abuse Treatment Utilization,” International Journal of Offender Therapy and Comparative Criminology 55, no. 4 (2011): 567–586. 10.1177/0306624X10366012 PMC2923294 20463206

[3] F. Ali , J. Mende‐Gibson , S. Imtiaz , et al., “Mapping the Overdose Crisis in Ontario: Geographic Disparities in Opioid‐Related Harms and Services,” BMC Public Health 25, no. 1 (2025): 3935. 10.1186/s12889-025-25103-y PMC12616965 41233802

[4] https://www.ons.gov.uk/peoplepopulationandcommunity/birthsdeathsandmarriages/deaths/bulletins/deathsrelatedtodrugpoisoninginenglandandwales/2024registrations, accessed October 23, 2026.

[5] https://www.ons.gov.uk/peoplepopulationandcommunity/healthandsocialcare/healthinequalities/bulletins/characteristicsofwomenwithanendometriosisdiagnosisinengland/27march11to31december21, accessed October 23, 2026.

[6] P. Congdon , “Geographical Patterns in Drug‐Related Mortality and Suicide: Investigating Commonalities in English Small Areas,” International Journal of Environmental Research and Public Health 16, no. 10 (2019): 1831. 10.3390/ijerph16101831 PMC6572137 31126097

[7] https://www.health.org.uk/evidence‐hub/risk‐factors/inequalities‐in‐drug‐related‐deaths, accessed October 23, 2026.

[8] E. Von Elm , D. G. Altman , M. Egger , S. J. Pocock , P. C. Gøtzsche , and J. P. Vandenbroucke , “The Strengthening the Reporting of Observational Studies in Epidemiology (STROBE) Statement: Guidelines for Reporting Observational Studies,” Annals of Internal Medicine 147, no. 8 (2007): 573–577. 10.7326/0003-4819-147-8-200710160-00010 17938396

[9] E. Roberts , C. Copeland , K. Humphreys , and C. L. Shover , “Drug‐Related Deaths Among Housed and Homeless Individuals in the UK and the USA: Comparative Retrospective Cohort Study,” British Journal of Psychiatry 223, no. 6 (2023): 562–568. 10.1192/bjp.2023.111 PMC10727910 37665046

[10] https://www.gov.uk/after‐a‐death/when‐a‐death‐is‐reported‐to‐a‐coroner, accessed October 23, 2026.

[11] https://www.ons.gov.uk/methodology/geography/geographicalproducts/ruralurbanclassifications/2021ruralurbanclassification, accessed October 23, 2026.

[12] https://geoportal.statistics.gov.uk/documents/c8e8e6db38e04cb8937569d74bce277a/about, accessed October 23, 2026.

[13] D. Sharpe , “Your Chi‐Square Test Is Statistically Significant: Now What?,” Practical Assessment, Research & Evaluation 20 (2015): 1–10.

[14] https://www.ons.gov.uk/methodology/classificationsandstandards/otherclassifications/thenationalstatisticssocioeconomicclassificationnssecrebasedonsoc2010, accessed October 23, 2026.

[15] A. Bukten , M. R. Stavseth , S. Skurtveit , A. Tverdal , J. Strang , and T. Clausen , “High Risk of Overdose Death Following Release From Prison: Variations in Mortality During a 15‐Year Observation Period,” Addiction 112, no. 8 (2017): 1432–1439. 10.1111/add.13803 28319291

[16] R. Barry , J. Rehm , C. de Oliveira , P. Gozdyra , and P. Kurdyak , “Rurality and Risk of Suicide Attempts and Death by Suicide Among People Living in Four English‐Speaking High‐Income Countries: A Systematic Review and Meta‐Analysis,” Canadian Journal of Psychiatry 65, no. 7 (2020): 441–447. 10.1177/0706743720902655 PMC7298579 31994903

[17] M. Prazak , R. Bacigalupi , and S. C. Hamilton , “Rural Suicide: Demographics, Causes, and Treatment Implications,” Community Mental Health Journal 61, no. 1 (2025): 66–75. 10.1007/s10597-024-01327-x PMC11703894 39102059

[18] R. Barry , J. Rehm , C. de Oliveira , P. Gozdyra , S. Chen , and P. Kurdyak , “The Relationship Between Rurality, Travel Time to Care and Death by Suicide,” BMC Psychiatry 23, no. 1 (2023): 345. 10.1186/s12888-023-04805-w PMC10189916 37198612

[19] L. R. Metsch and C. B. McCoy , “Drug Treatment Experiences: Rural and Urban Comparisons,” Substance Use & Misuse 34, no. 4–5 (1999): 763–784. 10.3109/10826089909037242 10210104

[20] https://cpe.org.uk/quality‐and‐regulations/market‐entry/rural‐issues/, accessed October 23, 2026.

[21] S. Chen , D. Mais , and C. S. Copeland , “Comparison of Office for National Statistics (ONS) and National Programme on Substance Use Mortality (NPSUM) Data Suggests That Opioid‐Related Deaths in England & Wales Have Been Systematically Underestimated,” International Journal of Drug Policy 145 (2025): 104976. 10.1016/j.drugpo.2025.104976 40886523

